# Epidemiology and risk factors for avascular necrosis in childhood systemic lupus erythematosus in a Taiwanese population

**DOI:** 10.1038/s41598-020-71923-w

**Published:** 2020-09-23

**Authors:** Hsin-Lin Tsai, Jei-Wen Chang, Jen-Her Lu, Chin-Su Liu

**Affiliations:** 1 Division of Pediatric Surgery, Department of Surgery, Taipei Veterans General Hospital, Taipei, Taiwan, ROC; 2 Faculty of Medicine, School of Medicine, National Yang-Ming University, Taipei, Taiwan, ROC; 3 Department of Pediatrics, Taipei Veterans General Hospital, No. 201, Sec. 2, Shipai Rd., Beitou District, Taipei, 11217 Taiwan, ROC

**Keywords:** Musculoskeletal system, Rheumatic diseases, Risk factors

## Abstract

Childhood-onset systemic lupus erythematosus (SLE) is associated with greater disease activity, more aggressive course, and high rates of organ damage. The prolonged use of corticosteroids in childhood SLE contributes to increased morbidity, including avascular necrosis (AVN). We conducted this retrospective study using claims data from the Taiwan National Health Insurance Research Database, enrolling 1,472 children with newly-diagnosed SLE between 2005 and 2013. The mean age at the diagnosis of SLE was 15.5 ± 3.3 years, and the female to male ratio was 6.2:1. Thirty-nine patients (2.6%) developed symptomatic AVN during a mean follow-up of 4.6 ± 2.5 years. In multivariate analysis, the risk of AVN was higher in the patients with a daily prednisolone dose between 7.5 mg and 30 mg (HR 7.435, 95% CI 2.882–19.178, p < 0.001) and over 30 mg (HR 9.366, 95% CI 2.225–39.418, p = 0.002) than in those with a dose ≤ 7.5 mg/day. In addition, AVN was inversely correlated with the use of hydroxychloroquine > 627 days (HR 0.335, 95% CI 0.162–0.694, p = 0.003). In conclusion, high daily doses of prednisolone were associated with a significant risk of AVN, whereas the use of hydroxychloroquine > 627 days conferred an advantage. We suggest that the judicious use of corticosteroids combined with hydroxychloroquine might be a promising preventive strategy for AVN.

## Introduction

Systemic lupus erythematosus (SLE) is an autoimmune disease with a wide range of disease manifestations. It can affect almost every organ system in the body including the kidneys, skin, blood cells, joints and nervous system. Approximately 15–20% of patients with lupus are diagnosed in childhood or adolescence. Childhood SLE is characterized by higher disease activity at presentation, and patients with childhood SLE are more prone to develop a more aggressive, unpredictable, and relapsing–remitting disease course during puberty^1,2^. Consequently, patients with childhood SLE need long-term and more intense corticosteroid and immunosuppressive treatment than those with adult-onset SLE to control disease exacerbations. Thus, childhood SLE is associated with increased treatment-related damage, morbidity and mortality.

Avascular necrosis (AVN) is a disorder in which bone death occurs due to interrupted blood supply, and it can result in significant morbidity and mortality. It typically occurs in patients aged 20–50 years, but rarely in children. Moreover, AVN is a well-known complication in patients with SLE^3^, and it occurs most frequently in patients treated with corticosteroids^4^. The pathophysiology of AVN is not completely understood. Although the use of corticosteroids has been reported to be a major risk factor for the development of AVN^5^, previous studies have demonstrated that AVN can occur in patients not treated with corticosteroids^6,7^. Nevertheless, AVN has been reported to occur more frequently in patients with SLE than in those with other illnesses requiring the administration of systemic corticosteroids, suggesting that SLE-specific risk factors may be associated with the development of AVN.

Most studies investigating the association between AVN and SLE have focused on adults rather than children. Advances in treatment strategies for childhood SLE have improved survival over the past decades, and therefore the prevention and management of morbidity and the long-term adverse effects of SLE treatments such as AVN have become increasingly important. The prevalence of SLE is higher among people of Asian and African descent^8^, and race/ethnicity remains a key determinant of disease expression in both adult and childhood SLE. However, most previous studies regarding AVN in childhood SLE have been limited to Western countries, and few studies have included Taiwanese patients. Therefore, the aim of the present study was to assess associations among clinical factors and commonly used medications for SLE and AVN in childhood SLE in Taiwan using the National Health Insurance Research Database (NHIRD), with a focus on corticosteroids.

## Results

### Demographic data and clinical characteristics

The demographic characteristics and comorbid conditions of the patients in the AVN and non-AVN groups are shown in Table 1. The study cohort consisted of 1,472 children newly diagnosed with childhood-onset SLE from 2005–2013. Of these 1,472 children, 1,268 (86.1%) were female, with a female to male ratio of 6.2:1. The mean age at diagnosis of SLE was 15.5 ± 3.3 years. Compared to the non-AVN group, the patients with AVN were older at the onset of SLE, however the difference was not statistically significant. The diagnosis of childhood-onset SLE was most common between the ages of 13–20 years, with 1,194 patients (81.1%) being diagnosed at an age ≥ 13 years.

**Table 1:** Demographic characteristics and clinical features of childhood SLE.

	Total (n = 1,472)	AVN (n = 39)	Non-AVN (n = 1,433)	p value
Sex (female/male)	1,268/204	37/2	1,231/202	0.110
Age at onset of SLE (years)	15.5 ± 3.3	16.2 ± 2.8	15.4 ± 3.3	0.158
Comorbidities				
Hypertension	282 (19.2%)	8 (20.5%)	274 (19.1%)	0.827
Hyperlipidemia	157 (10.7%)	7 (17.9%)	150 (10.5%)	0.180
LN	493 (33.5%)	19 (48.7%)	474 (33.1%)	0.041*
Proteinuria	166 (11.3%)	4 (10.3%)	162 (11.3%)	1
Nephrotic syndrome	244 (16.6%)	10 (25.6%)	234 (16.3%)	0.123
Treatment				
Cyclophosphamide	424 (28.8%)	18 (46.2%)	406 (28.3%)	0.015*
DMARDs	1,364 (92.7%)	33 (84.6%)	1,331 (92.9%)	0.061
Sulfasalazine	36 (2.4%)	1 (2.6%)	35 (2.4%)	1
Azathioprine	865 (58.8%)	26 (66.7%)	839 (58.5%)	0.310
Hydroxychloroquine	1,267 (86.1%)	30 (76.9%)	1,237 (86.3%)	0.094
Cumulative duration of hydroxychloroquine use (day)	797.2 ± 724.8	544.2 ± 580.1	804.1 ± 727.2	0.009*
History of corticosteroids use	1,406 (95.5%)	39 (100%)	1,367 (95.4%)	0.416
Mean daily dose of prednisolone (mg)	11.6 ± 16.4	17.0 ± 11.0	11.5 ± 16.5	0.037*
Mean daily dose of prednisolone-equivalent doses (mg)				< 0.001*
Low dose (≤ 7.5 mg/day)	702 (47.7%)	5 (12.8%)	697 (48.6%)	
Medium dose (7.5–30 mg/day)	684 (46.5%)	31 (79.5%)	653 (45.6%)	
High dose (> 30 mg/day)	86 (5.8%)	3 (7.7%)	83 (5.8%)	
Total cumulative dose of prednisolone (g)	16.3 ± 16.7	16.3 ± 14.0	16.3 ± 16.7	0.988
Total cumulative dose of prednisolone (g)				0.477
0–5 g	414 (28.1%)	9 (23.1%)	405 (28.3%)	
> 5 g	1,058 (71.9%)	30 (76.9%)	1,028 (71.7%)	

AVN, avascular necrosis; DMARDs, disease-modifying antirheumatic drugs; LN, lupus nephritis; SLE, systemic lupus erythematosus.

*p < 0.05.

**Figure 1:**
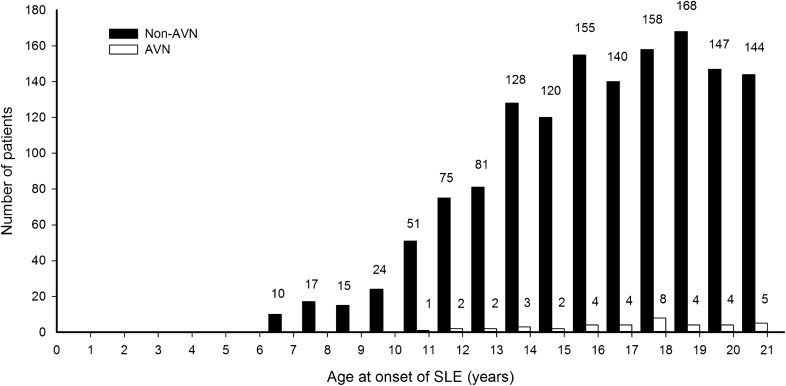
Age distribution at SLE onset in children with and without AVN. AVN did not develop in any patient who was younger than age 10 years at the time of SLE onset.

**Figure 2:**
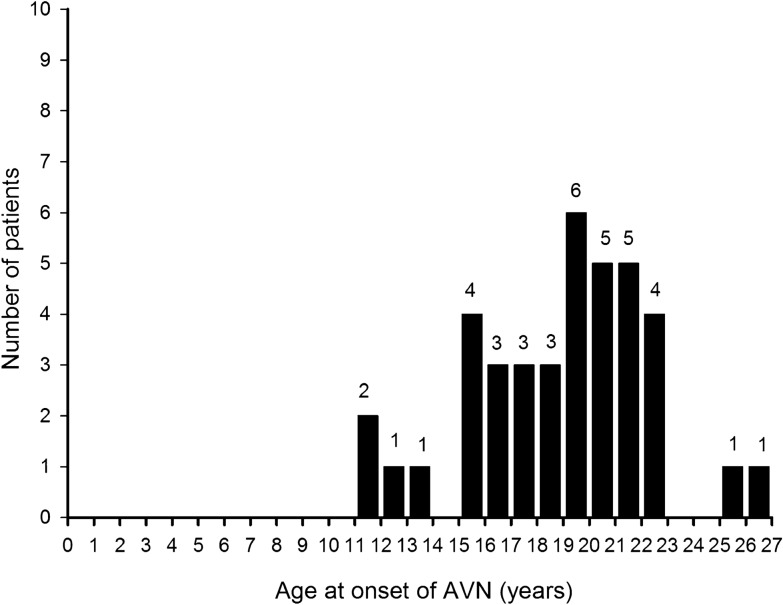
Age distribution at onset of AVN in children with SLE. AVN did not develop in any patient who was younger than age 11 years.

Overall, 39 children (2.6%) including 37 girls and two boys developed AVN after an average follow-up of 4.6 ± 2.5 years. There was clear female predominance in the AVN group with a ratio of 18.5:1, although the difference was not statistically significant. The mean age at diagnosis of AVN was 19.0 ± 3.5 years, with mean disease duration to AVN of 2.8 ± 1.9 years. Sixteen (41.0%) patients developed AVN within 2 years after the diagnosis of SLE, and the remaining 23 patients (59%) developed AVN 2–8 years after the onset of SLE. The age distribution at the onset of SLE in patients with and without AVN is shown in Fig. 1. All of the children with AVN were diagnosed with SLE after 10 years of age. Figure 2 shows the age at onset of AVN. The youngest patient in whom AVN developed was 11.4 years old, and two (5.1%) of the AVN patients were < 12 years old. Four hundred and ninety-three (33.5%) of the patients had lupus nephritis (LN), and LN was significantly associated with the development of AVN (48.7% vs. 33.1%, p = 0.041). The most common comorbid conditions in the children with SLE were hypertension (19.2%), nephrotic syndrome (16.6%), proteinuria (11.3%) and hyperlipidemia (10.7%). The patients with AVN had higher rates of coexisting hypertension, hyperlipidemia and nephrotic syndrome. However, there were no significant differences in hypertension, hyperlipidemia and nephrotic syndrome between the AVN and non-AVN groups.

### DMARDs, immunosuppressives, and corticosteroids

Overall, 1,364 (92.7%) patients received disease-modifying antirheumatic drugs (DMARDs), of which hydroxychloroquine was the most commonly used (86.1%). The AVN group had fewer prescriptions of hydroxychloroquine (76.9% vs. 86.3%) but more prescriptions of sulfasalazine and azathioprine than those without AVN, although none of the differences were statistically significant. The cumulative duration of hydroxychloroquine exposure in the AVN group was significantly shorter than that in the non-AVN group (544.2 ± 580.1 vs.804.1 ± 727.2 days, p = 0.009). Significantly more patients with AVN received cyclophosphamide compared to those without AVN (46.2% vs. 28.3%, p = 0.015). Most of the patients (1,406, 95.5%) were prescribed with systemic corticosteroids during the study period, and all of the patients who developed AVN received steroid treatment at some point during their disease course. The mean daily dose of prednisolone was significantly higher in the SLE patients with AVN (p = 0.037), however the difference in cumulative dose of prednisolone did not reach statistical significance.

### Univariate and multivariate analyses of risk factors for the development of AVN

Table 2 presents the results of the univariate and multivariate analyses for associations between AVN and demographic and clinical factors. In univariate analysis, AVN was associated with a daily prednisolone dose between 7.5 mg and 30 mg and > 30 mg with hazard ratios (HRs) of 6.493 (95% confidence interval (CI) 2.525–16.698, p < 0.001) and 9.609 (95% CI 2.287–40.372, p = 0.002), respectively. Among the prescribed DMARDs, the use of hydroxychloroquine and a cumulative duration of hydroxychloroquine use > 627 days were negatively correlated with AVN with HRs of 0.426 (95% CI 0.202–0.897, p = 0.025) and 0.362 (95% CI 0.189–0.693, p = 0.002), respectively. Sex, age at SLE onset, comorbidities (hypertension and hyperlipidemia), presence of LN, proteinuria or nephrotic syndrome, use of sulfasalazine, azathioprine or cyclophosphamide, and a higher cumulative dose of corticosteroids were not correlated with AVN. In multivariate analysis, the risk of AVN was higher in the patients with a higher daily prednisolone dose between 7.5 mg and 30 mg (HR 7.435, 95% CI 2.882–19.178, p < 0.001) and > 30 mg (HR 9.366, 95% CI 2.225–39.418, p = 0.002) than in those with a dose ≤ 7.5 mg/day. AVN remained inversely correlated with the use of hydroxychloroquine > 627 days (HR 0.335, 95% CI 0.162–0.694, p = 0.003).

**Table 2:** Univariate and multivariate Cox regression analysis of risk factors associated with AVN in childhood SLE.

	Univariate analysis		Multivariate analysis	
	HR (95% CI)	p value	HR (95% CI)	p value
Age at onset of SLE (years)				
6–12	1	Reference		
13–20	1.553 (0.607–3.970)	0.358		
Female	3.050 (0.735–12.656)	0.124		
Comorbidities				
Hypertension	0.935 (0.429–2.035)	0.865		
Hyperlipidemia	1.612 (0.711–3.654)	0.253		
LN	1.637 (0.873–3.070)	0.124		
Proteinuria	0.798 (0.284–2.246)	0.669		
Nephrotic syndrome	1.531 (0.745–3.144)	0.246		
Treatment				
Cyclophosphamide	1.677 (0.891–3.157)	0.109		
DMARDs				
Sulfasalazine	0.893 (0.123–6.503)	0.911		
Azathioprine	1.124 (0.577–2.190)	0.731		
Hydroxychloroquine	0.426 (0.202–0.897)	0.025*	0.764 (0.334–1.749)	0.524
Cumulative duration of hydroxychloroquine use > 627 days	0.362 (0.189–0.693)	0.002*	0.335 (0.162–0.694)	0.003*
Mean daily dose of prednisolone-equivalent doses				
Low dose (≤ 7.5 mg/day)	1	Reference	1	Reference
Medium dose (7.5–30 mg/day)	6.493 (2.525–16.698)	< 0.001*	7.435 (2.882–19.178)	< 0.001*
High dose (> 30 mg/day)	9.609 (2.287–40.372)	0.002*	9.366 (2.225–39.418)	0.002*
Total cumulative dose of prednisolone > 5 g	0.789 (0.373–1.667)	0.534		

AVN, avascular necrosis; DMARDs, disease-modifying antirheumatic drugs; LN, lupus nephritis; SLE, systemic lupus erythematosus.

*p < 0.05.

## Discussion

Childhood-onset SLE comprises only 15–20% of all SLE cases. Similar to adult SLE, childhood SLE occurs more frequently in females, although to a lesser degree compared to adult-onset SLE. The epidemiological findings of the current study with regards to childhood SLE are consistent with previously published studies. Childhood SLE is clinically and serologically different from adult SLE^9,10^. Patients with childhood SLE tend to have higher disease activity at presentation, a more aggressive clinical course, increased rate of organ damage, and lower remission rate. In addition, patients with childhood SLE have been reported to have a higher frequency of nephritis^11^, hematological manifestations and central nervous system involvement. In childhood SLE, prolonged high-dose systemic corticosteroid therapy and additional immunosuppressive drugs are required for the majority of patients. Despite a general lack of comorbid conditions, previous studies have shown that patients with childhood SLE are at a greater risk of mortality compared to patients with adult SLE, and especially those with LN^12,13^. With earlier recognition of the disease, increased availability of new immunosuppressants and improved intensive care, the 5-year and 10-year survival rates for childhood SLE have improved considerably in the last decade^14^. As survival rates for childhood SLE have improved, prevention and early treatment of morbidities resulting from disease activity and adverse effects of treatment, and especially corticosteroids and immunosuppressants, have become increasingly important. Long-term survivors face substantial risks of morbidity such as recurrent infections, hypertension, dyslipidemia, atherosclerosis, growth failure, and AVN.

AVN is the death of bone tissue due to interruption of the blood supply. Several population-based studies have reported that AVN is a common complication in adult SLE, however large-scale research in children is limited. To the best of our knowledge, this is the largest pediatric study to date on the association between SLE and AVN. Overall, 2.6% of our patients demonstrated evidence of symptomatic AVN, which is lower than in previous studies (ranging from 5.4 to 40%)^15–21^ but similar to the 3.1% reported by Bogmat et al.^22^ Affected individuals can be asymptomatic in the early stages of disease, and are detected by magnetic resonance imaging. Therefore, the true prevalence rate in our cohort is likely to be higher as only symptomatic cases who sought medical care were identified. However, as AVN progresses, most people will experience joint pain and limited movement. When the diagnosis of AVN is made early, symptoms can be eased with medications. Nakamura et al.^23^ and Yang et al.^24^ reported that the rate of AVN was lower prior to the onset of puberty than in adolescents or adult patients. Possible mechanisms to explain these trends of female predominance and higher risk after puberty include estrogen, which is assumed to be an important factor in SLE, and sex hormonal changes during puberty, which can influence disease activity and the course of childhood SLE. Furthermore, estrogen during puberty can increase the procoagulant effects in contrast to testosterone which inhibits platelet aggregation through increased nitrogen monoxide release in endothelial cells. Furthermore, red to yellow marrow conversion^25^ and pubertal growth spurts can lead to excessive metabolic activity and increased oxygen consumption in growth plates and bones, and along with increased physical activity can contribute to increased susceptibility to ischemic injury and then AVN in pubertal children. There was a strong female predominance, and the majority of the AVN episodes were diagnosed when the patients were older than 12 years in the present study. However, our data did not show an association between AVN and sex or age at the onset of SLE.

The exact pathogenesis of AVN in SLE has yet to be definitively elucidated, however multiple factors have been proposed. Corticosteroid usage has been reported to be a major risk factor for the development of AVN in adults^26^. The average daily dose and highest dose of corticosteroids as well as total cumulative dose of corticosteroids and pulse therapy have also been reported to be associated with AVN^3^. Consistent with these studies, we also found that the vast majority of the patients with childhood-onset SLE received corticosteroid treatment. Moreover, all of the AVN patients received corticosteroids and a significantly higher mean daily dose. Those taking a medium (7.5–30 mg/day) or high (> 30 mg/day) daily dose of prednisolone had increased HRs for AVN of 7.435 and 9.366 fold, respectively. The mechanisms by which corticosteroids induce AVN have yet to be fully elucidated. Chronic corticosteroid use is known to induce fat cell hypertrophy with increased intraosseous pressure, fat embolization, intravascular coagulation and osteocyte apoptosis^27,28^. Corticosteroids also regulate local blood flow by modulating vasoactive substances such as nitric oxide, endothelin-1, noradrenalin and bradykinin^28^. Similar to other studies, the present study showed that the patients with other diseases such as nephrotic syndrome treated with chronic corticosteroids did not have an increased risk of AVN. AVN has rarely been reported in SLE patients who did not receive corticosteroids, suggesting that corticosteroids may not be the only risk factor associated with the development of AVN, and that SLE itself may contribute to the development of AVN. Several potential risk factors for AVN have been reported^3^, including disease activity^29^, vasculitis^5^, serositis^5,30^, cytopenias^7^, arthritis^31^, Raynaud's phenomenon^5^, neuropsychiatric symptoms and the presence of antiphospholipid antibodies^32^. Ongoing SLE disease activity may lead to damage including to bone tissue, and the prolonged use of high-dose corticosteroids to control disease activity may further increase the risk of developing AVN. Immunosuppressive drugs are widely used as steroid-sparing drugs for patients with severe disease activity. In addition to corticosteroids, several studies have reported an association between immunosuppressive agents, especially cyclophosphamide, and the development of AVN^3^. As with corticosteroids, the use of cyclophosphamide can be a surrogate for more severe disease activity, which may be a pathogenetic factor for AVN. In the present study, the patients taking cyclophosphamide were significantly associated with the development of AVN. However, the use of cyclophosphamide did not reach significance in the univariate and multivariate Cox regression analyses. In contrast to other series, our study did not find an association between azathioprine and AVN. Intentional and unintentional non-adherence to medications is common in children and adolescents with rheumatic diseases, however we could not analyze compliance with taking prescribed medications in this study. The doses of corticosteroids and immunosuppressive agents were calculated based on the assumption of good overall adherence, and may therefore be inaccurate for some patients.

Prior studies have reported that racial/ethnic disparities exist in the incidence, disease course and outcomes of adult SLE. Similar to adult SLE, childhood SLE has also been reported to be more severe in non-white populations, especially African American, Asian, Hispanic and Aboriginal populations^33,34^. Childhood SLE carries a worse prognosis than in adults, and the reported estimated prevalence of LN ranges from 29 to 80.8% in pediatric SLE cohorts^35,36^. In our study, 33.5% of the cases had LN, which is comparable to the findings of Alsaeid et al.^35^ who reported that 29% of the children in their study had renal biopsy-proven LN. Most previous studies have reported that renal involvement is a risk factor for AVN in patients with SLE^24,37^. Our results showed that the children with AVN had a significantly higher prevalence of LN (48.7% vs. 33.1%; p = 0.041). However, no significant association was found in univariate or multivariate Cox regression analysis.

The complex interactions between disease activity, chronic inflammation, cytokines, renal disease, corticosteroids, cyclosporine and diet contribute to the development of metabolic syndrome in SLE patients. Therefore, patients with childhood SLE are at a high risk of developing premature atherosclerosis leading to occlusion of arteries by thrombosis. Some authors have suggested that hypertension^3, 21^, diabetes mellitus and hyperlipidemia are risk factors for AVN, and that lipid-lowering agents such as statins can decrease the risk of corticosteroid-related AVN through reducing lipid levels in blood as well as anti-inflammatory responses of endothelial cell^38^. In the present study, hypertension and hyperlipidemia were not associated with AVN. Hydroxychloroquine is commonly used in the management of SLE to prevent disease flares and to treat mild disease activity, and it has also been shown to have antithrombotic and lipid-lowering properties^39^. However, the beneficial effect of hydroxychloroquine in AVN is still controversial^40,41^. We found that the use of hydroxychloroquine > 627 days had a protective effect against the development of AVN. Bone marrow adipocyte hyperplasia and hypertrophy are the principal mechanisms involved in the onset and progression of AVN. In addition, autophagy activation has recently been reported to play an important role when adipocytes undergo differentiation and hyperplasia^42,43^. We hypothesized that in addition to immunomodulatory activity, hydroxychloroquine may prevent AVN through autophagy suppression in bone marrow adipose tissue. However, the precise functions of hydroxychloroquine in AVN remain to be elucidated in future studies.

AVN in children with lupus remains understudied, and most published studies of AVN are based on single-institution investigations. This is the first large-scale nationwide population-based cohort study of AVN in patients with childhood SLE. The major strengths of the current study include that the identification of SLE was highly reliable through the use of catastrophic illness certificates, and the population-based design. However, there are several important limitations. First, asymptomatic AVN may not have been identified in this study. Second, patient data on serologies, laboratory studies, clinical symptoms and major organ damage due to disease activity are unavailable in the NHIRD. Third, personal data including height, body weight, weight gain after the use of corticosteroids, exercise, alcohol use, and self-paid drugs to control disease activity such as mycophenolate mofetil are also unavailable in the NHIRD. Therefore, the cumulative and mean daily doses of corticosteroids were not calculated as per kg of body weight due to the limitation and characteristics of the NHIRD.

In conclusion, AVN occurred in 2.6% of the children with SLE in this study. A high daily dose of prednisolone was associated with a significant risk of AVN, whereas the use of hydroxychloroquine conferred an advantage. Recognizing the risk factors and concomitant hydroxychloroquine therapy remain the most effective ways of preventing morbidities caused by corticosteroid use. Further prospective studies are needed to clarify the risk factors for the development of AVN in childhood SLE.

## Methods

### Data source

This study was based on the NHIRD, a longitudinal health insurance database which is provided by the Taiwan National Health Research Institute for research purposes. Taiwan launched the single-payer National Health Insurance (NHI) program in March 1995, and more than 99% of the population (approximately 23 million people) are currently enrolled. The NHIRD includes all registry and claims data from the NHI system, including demographic data, detailed data of prescription drugs, and orders of ambulatory and inpatient care. All retrieved data from the NHIRD are de-identified and encrypted before the database is released for research use. In the NHIRD, International Classification of Diseases, Ninth Revision, Clinical Modification (ICD-9-CM) codes are used to define diseases.

In Taiwan, patients with certain autoimmune diseases such as SLE, systemic sclerosis, rheumatoid arthritis, Sjogren’s syndrome, pemphigus and inflammatory bowel disease can apply for a catastrophic illness certificate and are exempt from the usual co-payments for inpatient or outpatient care related to that condition within the certificate’s validity period. The Bureau of NHI routinely validates the diagnoses based on a review of the medical records, laboratory studies and imaging studies of the patients who apply for catastrophic illness certification. The accuracy and reliability of the data of these patients with catastrophic illnesses are therefore very high. This study was approved by the Institutional Review Board of Taipei Veterans General Hospital, Taiwan (Approval Number: 2016-08-013CC) and the requirement for informed consent was waived. This study was carried out in accordance with relevant guidelines and regulations.

### Study cohort

Childhood SLE was defined if the diagnosis of SLE was made at an age of 6–20 years. To validate the diagnosis of SLE, patients with a new diagnosis of SLE between January 1, 2005 and September 30, 2013 were identified from catastrophic illness certificates in the NHIRD according to ICD-9-CM codes 710.0 and 695.4. The date of SLE onset was defined as the date of application for a catastrophic illness certificate. All enrolled index cases were followed up from the date of diagnosis with SLE until the diagnosis of AVN, death, or December 31, 2013 (end of the study period) whichever occurred first.

### Outcome measures

The outcome was defined as the development of AVN. The study subjects were considered to have AVN if they had received medical care at outpatient visits or received inpatient services for a diagnosis of AVN (ICD-9-CM codes 733.4X) after the diagnosis of SLE.

### Potential confounding comorbidities

The demographic data of the patients including their age at onset of SLE, sex, and age at the diagnosis of AVN were extracted. The patients were classified into two age subgroups based on their age at SLE onset: pediatric (6–12 years old) and adolescent (13–20 years old). Comorbidities such as hypertension (ICD-9-CM codes 401–405), hyperlipidemia (ICD-9-CM code 272), proteinuria (ICD-9-CM code 791.0), nephrotic syndrome (ICD-9-CM code 581.XX), and LN (ICD-9-CM code 583.81) were identified from claims data based on either one inpatient diagnosis or two outpatient diagnoses.

### Corticosteroid, DMARDs and cyclophosphamide treatment

Data on the use of corticosteroids, DMARDs and immunosuppressive medications after the first diagnosis of SLE until the diagnosis of AVN, death, or the end of the study period, whichever occurred first, were analyzed. We used NHI drug codes and Anatomical Therapeutic Chemical (ATC) codes to identify the patients who were prescribed with any corticosteroids, DMARDs and cyclophosphamide. The oral corticosteroids (outpatients and inpatients) and intravenous corticosteroids (inpatients) assessed in this study included betamethasone (ATC code H02AB01), dexamethasone (ATC code H02AB02), methylprednisolone (ATC code H02AB04), paramethasone (ATC code H02AB05), prednisolone (ATC code H02AB06), triamcinolone (ATC code H02AB08), hydrocortisone (ATC code H02AB09) and cortisone (ATC code H02AB10). The doses of all corticosteroids were converted to the equivalent dose of prednisolone. Mean daily prednisolone equivalent dosage was categorized into three levels according to a modification of the European League against Rheumatism Standing Committee classification of daily prednisolone dose: low dose (≤ 7.5 mg/day), medium dose (7.5–30 mg/day), and high dose (> 30 mg/day)^44^. The DMARDs analyzed in this study included sulfasalazine (ATC code A07EC01), azathioprine (ATC code L04AX01) and hydroxychloroquine (ATC code P01BA02). The cumulative duration of hydroxychloroquine use was calculated. The use of cyclophosphamide (ATC code L01AA01) was also extracted for analysis. The immunosuppressants mycophenolate mofetil and cyclosporine were not analyzed as these two drugs are not covered by the NHI program for the treatment of SLE.

### Statistical analysis

To compare characteristics between SLE patients with and without AVN, the Student’s *t* test was used for continuous data and the chi-square or Fisher’s exact test for categorical variables. A Cox proportional hazards model was used to calculate HRs and 95% CIs to determine independent predictors for AVN. All pertinent variables including age at onset of SLE, sex, mean daily corticosteroid dosage, DMARDs and comorbidities were first examined individually in univariate analyses. Variables with p < 0.05 in univariate analysis for an association with the development of AVN were entered into multivariate analysis as covariates. All procedures were performed using SPSS version 19.0. A p value < 0.05 was considered to indicate statistical significance.
